# Ectopic *KIT* Copy Number Variation Underlies Impaired Migration of Primordial Germ Cells Associated with Gonadal Hypoplasia in Cattle (*Bos taurus*)

**DOI:** 10.1371/journal.pone.0075659

**Published:** 2013-09-26

**Authors:** Heli Venhoranta, Hubert Pausch, Michal Wysocki, Izabela Szczerbal, Reetta Hänninen, Juhani Taponen, Pekka Uimari, Krzysztof Flisikowski, Hannes Lohi, Ruedi Fries, Marek Switonski, Magnus Andersson

**Affiliations:** 1 Department of Production Animal Medicine, University of Helsinki, Saarentaus, Finland; 2 Chair of Animal Breeding, Technische Universität München, Freising-Weihenstephan, Germany; 3 Department of GeneticsandAnimal Breeding, University of Life Sciences, Poznań, Poland; 4 Department of Veterinary Biosciences, Research Programs Unit, Molecular Neurology, University of Helsinki and Folkhälsan Research Institute, Helsinki, Finland; 5 Agrifood Research Finland, MTT, Biotechnology and Food Research, Jokioinen, Finland; 6 Chair of Livestock Biotechnology, Technische Universität München, Freising, Germany; Massachusetts General Hospital, United States of America

## Abstract

Impaired migration of primordial germ cells during embryonic development causes hereditary gonadal hypoplasia in both sexes of Northern Finncattle and Swedish Mountain cattle. The affected gonads exhibit a lack of or, in rare cases, a reduced number of germ cells. Most affected animals present left-sided gonadal hypoplasia. However, right-sided and bilateral cases are also found. This type of gonadal hypoplasia prevails in animals with white coat colour. Previous studies indicated that gonadal hypoplasia is inherited in an autosomal recessive fashion with incomplete penetrance. In order to identify genetic regions underlying gonadal hypoplasia, a genome-wide association study (GWAS) and a copy number variation (CNV) analysis were performed with 94 animals, including 21 affected animals, using bovine 777,962 SNP arrays. The GWAS and CNV results revealed two significantly associated regions on bovine chromosomes (BTA) 29 and 6, respectively (P=2.19 x 10^-13^ and P=5.65 x 10^-6^). Subsequent cytogenetic and PCR analyses demonstrated that homozygosity of a ~500 kb chromosomal segment translocated from BTA6 to BTA29 (Cs_29_ allele) is the underlying genetic mechanism responsible for gonadal hypoplasia. The duplicated segment includes the *KIT* gene that is known to regulate the migration of germ cells and precursors of melanocytes. This duplication is also one of the two translocations associated with colour sidedness in various cattle breeds.

## Introduction

Gonadal hypoplasia is characterised by aberrantly small and underdeveloped gonads. Fertility is generally disturbed when both gonads are hypoplastic. Different types of gonadal hypoplasia have been reported in several mammalian species, including cats, dogs [1], sheep [2], horses [3,4] and humans [5].

Hereditary gonadal hypoplasia is a frequent disorder in two Scandinavian cattle breeds, Northern Finncattle and Swedish Mountain cattle (also called Swedish Highland breed). The defect emerged in the early 20th century after pure-breeding of Swedish Mountain cattle was implemented [6,7]. A few decades later the incidence of gonadal hypoplasia increased heavily and clinical investigations were initiated. Comprehensive health control evaluations were implemented which reduced the incidence of gonadal hypoplasia from 17.3% for Swedish Mountain cattle born before 1937 to 7.3% for animals born between 1952 and 1954 [8]. The Northern Finncattle almost became extinct during the Second World War. Thus considerable introgression of genes from Swedish Mountain cattle took place during the last 65 years and likely introduced gonadal hypoplasia into the Northern Finncattle herd.

The incidence of gonadal hypoplasia in the mentioned breeds is equal in both sexes and comprehensive breeding experiments suggest a recessive mode of inheritance with incomplete penetrance [7]. Penetrance was estimated to be 0.5 and Eriksson [7] suggested that the incomplete penetrance is partly genetically determined.

Gonadal hypoplasia is unique in Northern Finncattle and Swedish Mountain cattle because it is mainly manifested on the left side. The proportions of left-, double- and right-sided gonadal hypoplasia are 82%, 15% and 3%, respectively [7]. Although the absolute numbers differ across studies, left-sided gonadal hypoplasia always predominates [9,10]. However, there is no evidence that the localization (left and/or right) of hypoplastic gonads is genetically determined in Northern Finncattle and Swedish Mountain cattle [7].

**Figure 1:**
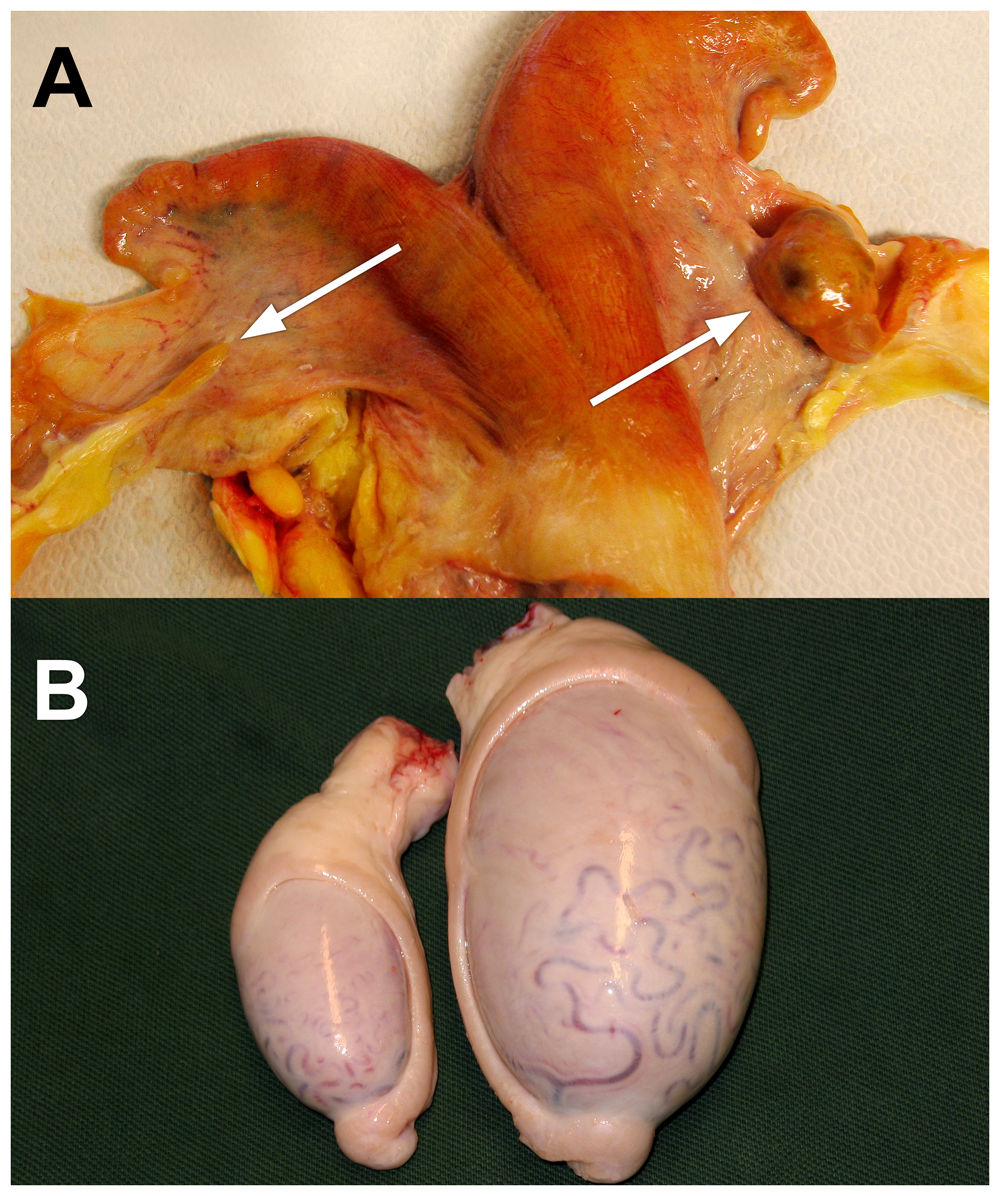
Left-sided gonadal hypoplasia. (A) Hypoplastic ovary (left arrow) and normal ovary (right arrow). (B) Hypoplastic testicle (left) and normal testicle (right).

The severity of gonadal hypoplasia varies considerably from total (Figure 1) to partial [6,7] and affected animals lack or have a reduced number of germ cells [6]. Bilaterally affected animals are often sterile and hypoplasia can also affect secondary sexual characteristics via impaired production of sexual hormones in females [7,9]. There are no reports that affected animals suffer from any other health problems.

Gonadal hypoplasia of Northern Finncattle and Swedish Mountain cattle is a congenital defect [7] and Settergren [6] found that the hypoplastic ovaries can already be identified at the foetal stage. Foetal testicles were not studied. Settergren [6] concluded that the reason for gonadal hypoplasia is a failure of the migration and synchronous mitotic divisions of primordial germ cells (PGC). Migration and proliferation of PGCs in the developing embryo is a complex process with various genes and signalling pathways involved [11,12].

Gonadal hypoplasia occurs only in animals that are 60-100% white coloured (Table S1) [6]. The coat colour of the Northern Finncattle and Swedish Mountain cattle varies from white to black or brown with numerous intermediates. White coat with pigmented ears and muzzle is the most common form, but spotted sides and coloured legs are also commonly found (Figure S1). Part of this colour variation is likely due the colour-sided pattern that is suggested to be caused by a semi-dominant gene symbolized as *Cs* [13,14]. Recently Durkin et al. [15] showed that colour sidedness is determined by two loci present on bovine chromosomes, (BTA) 29 and 6. The Cs_29_ allele in BTA29 resulted from a duplication and translocation event of a 492 kb segment of BTA6 including the *KIT* gene. The Cs_6_ allele residing on BTA6 is a result of a subsequent duplication and translocation event that moved back the segment comprising fused sequences of the BTA29 and BTA6 to the *KIT* locus in BTA6. It is expected that in both cases the dysregulation of the *KIT* gene leads to the colour sidedness [15].

The *KIT* gene encodes a type III receptor protein of the tyrosine kinase family. Several studies have shown that *KIT* protein is crucial for survival, proliferation and migration of melanocyte precursors and primordial germ cells (PGC) during embryogenesis [16-18]. *KIT* is also essential for regular development of hematopoietic cells [16,19]. In mice, mutations in *KIT* result in impaired pigmentation, reduced fertility or sterility, anaemia and deafness. These mutations are often pleiotropic (MGI:96677, http://www.informatics.jax.org/). *KIT* mutations also underlie coat colour variation in other mammals, e.g. pig [20,21] and horse [22]. In humans, *KIT* mutations cause piebaldism, mast cell disease and several types of tumour (*164920, http://omim.org/).

The aim of this study was to identify the genetic cause of the hereditary form of gonadal hypoplasia in Northern Finncattle and Swedish Mountain cattle. A genome-wide association study combined with a copy number variation (CNV) analysis was performed with high-density SNP arrays to pinpoint the genomic region. The results were confirmed with cytogenetic investigations, quantitative PCR and conventional PCR. We show that Swedish Mountain cattle and Northern Finncattle carry alleles that cause colour-sidedness [15] and that affected individuals are homozygous for the Cs_29_ allele that includes the ectopic *KIT* gene.

## Results

### A genome-wide association study maps congenital gonadal hypoplasia to BTA29

**Figure 2:**
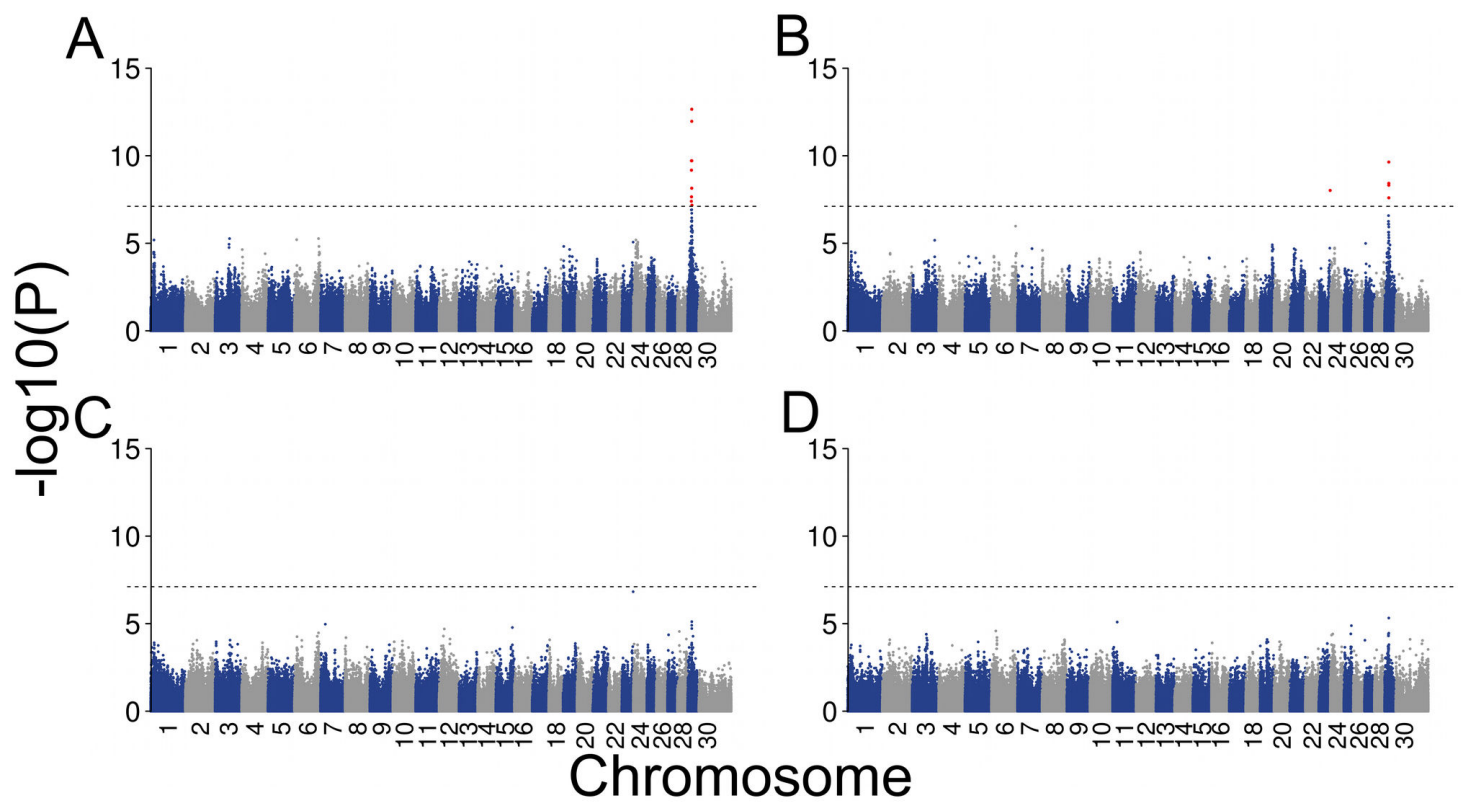
Association of 647,971 SNPs in four different case control scenarios. The Manhattan plots represent the -log_10_(P) values of association for 647,971 SNPs in four different case-control designs (Table 1). The red dots represent significantly associated SNPs (P < 7.71 x 10^-8^).

Gonadal hypoplasia in Northern Finncattle and Swedish Mountain cattle is hypothesized to result from pleiotropic effects of a white colour gene because most of the affected animals are predominantly white [6,23]. To account for both the different coat colour patterns and the affection status of the animals, four different case-control cohorts were established (Table 1) and allelic associations were performed. P-values below 7.71 x 10^-8^ (Bonferroni-corrected threshold for multiple testing) were considered to be significantly associated. A genome-wide association study (GWAS) with 20 unaffected predominantly black and brown animals and 21 affected predominantly white animals revealed 18 significantly associated SNPs on BTA29 between 17.69 Mb to 20.50 Mb (Figure 2A, Table S2). The SNP showing most significant association was BovineHD2900005672 (19,661,149 bp, P=2.19 x 10^-13^). The second GWAS, including all available unaffected animals (N=73) and all affected animals, yielded five highly significantly associated SNPs on BTA29 and one highly significantly associated SNP on BTA23 at 52,435,290 bp, (P=9.57 x 10^-9^) (Figure 2B). This SNP was considered to be a false positive result because there were no other significantly associated SNPs on BTA23. Moreover, re-genotyping the SNP using Sanger-sequencing revealed discrepant genotypes compared with the genotypes obtained with the Illumina BovineHD Bead chip in some samples. This indicated technical problems. Comparison of predominantly white -40 unaffected and 21 affected – animals revealed a suggestive association (Table S2). Similarly, GWAS in the fourth group (D) using 40 unaffected predominantly white animals and 20 unaffected predominantly coloured animals revealed a suggestive association (Table S2). However the SNPs of the last two GWAS analysis were not significantly associated after Bonferroni-correction (Figure 2C and 2D). Additionally, association analyses were repeated using a genotypic test. The results were in line with the allelic test, underlining the importance of the 17.69 Mb to 20.50 Mb area in BTA29 (Table S3). Collectively, the analysis of the four studied cohorts indicates that the BTA29 region is associated with gonadal hypoplasia and not only reflects different coat colour patterns. The genomic inflation factors for the groups ranged from 0.94 to 1.02 and indicate the absence of potential spurious associations.

**Table 1:** Organisation of four different case-control designs.

	Control cohort							Case cohort						
Gonadal hypoplasia	UNAFF			AFF				UNAFF			AFF			
Coat colour	W	B	U	W	B	U	total	W	B	U	W	B	U	total
Design A		20					20				21			21
Design B	40	20	13				73				21			21
Design C	40						40				21			21
Design D	40						40		20					20

Four different case-control cohorts were established (**A-D**). The coat colour of the animals was assessed as predominantly white (**W**), predominantly black/brown (**B**) and unknown (**U**). The gonadal hypoplasia status was assessed as a binary trait (affected (**AFF**) and unaffected (**UNAFF**)).

### CNV analysis identifies a translocated genomic region with the *KIT* gene

**Figure 3:**
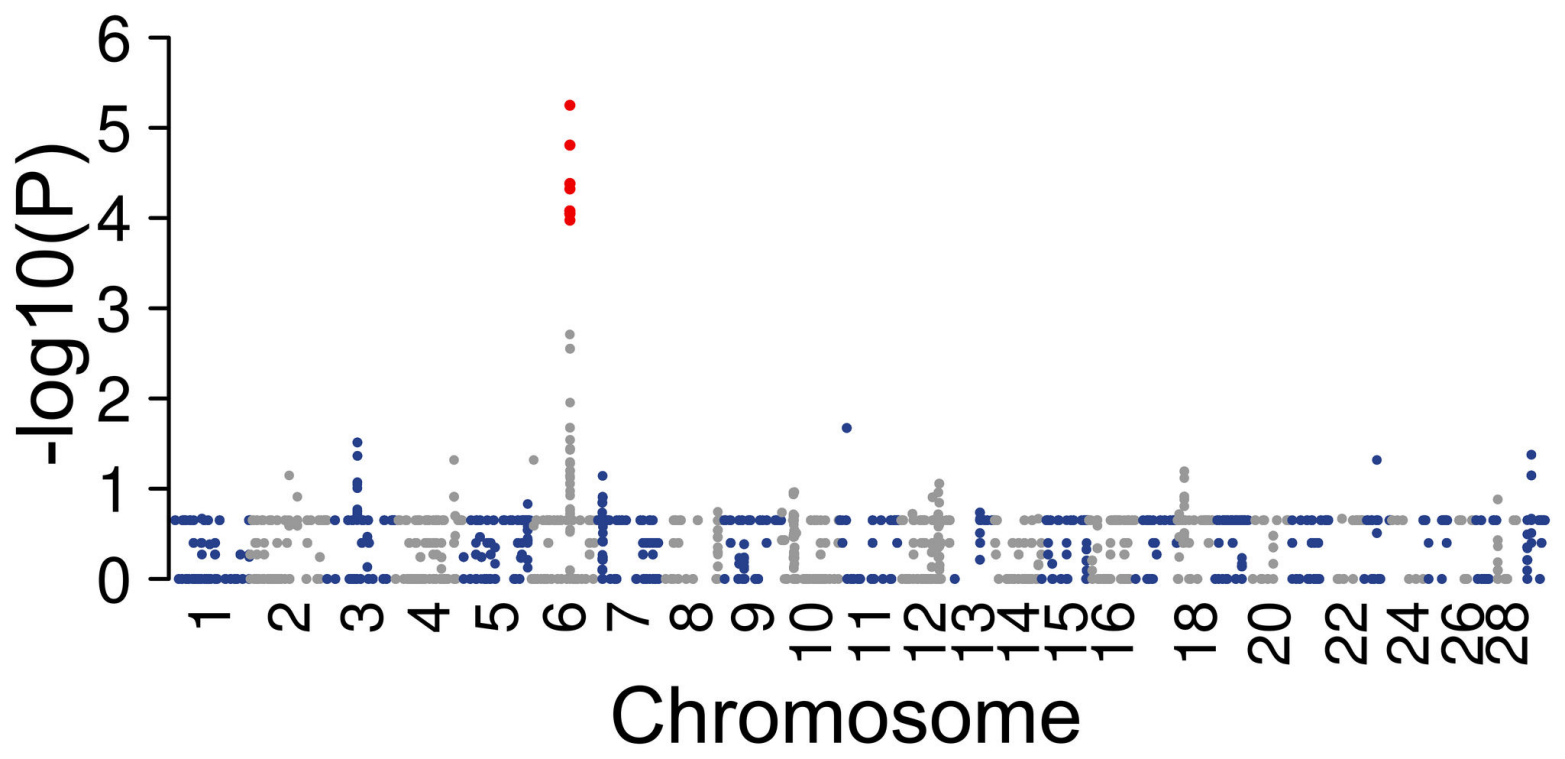
Association of 2101 autosomal CNVs with the affection status of 94 animals. The presence of CNV segments was compared in 21 cases and 73 controls using Fisher exact tests. The dots represent SNPs within the CNV segments. Red dots represent SNPs in significantly overrepresented CNVs in cases vs. controls (P < 2.38 x 10^-5^).

**Figure 4:**
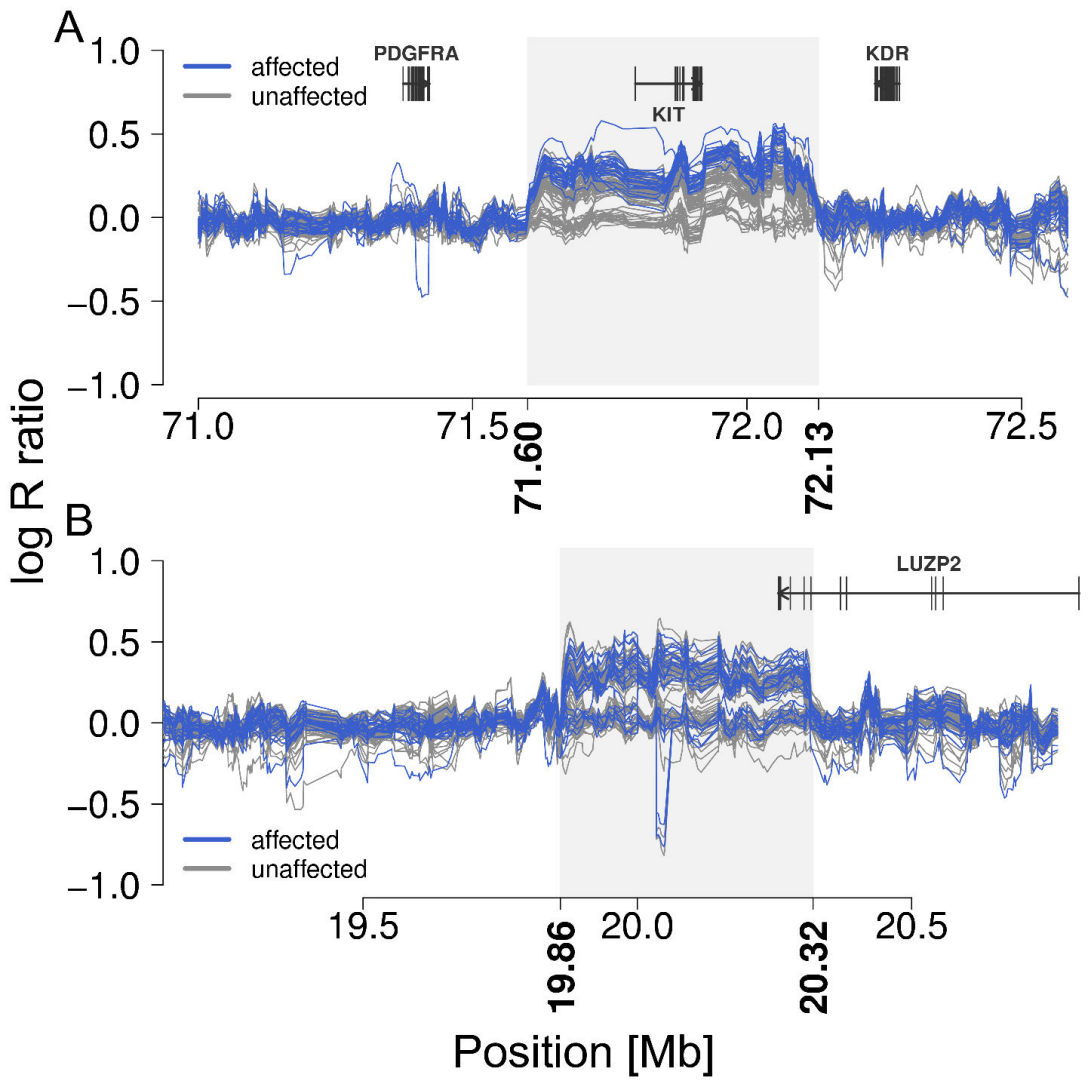
Schematic view of two CNV segments on BTA6 and BTA29. The figures display 5-SNP-sliding-window log R ratios in 75 unaffected (grey) and 21 affected (blue) animals on BTA6 (A) and BTA29 (B). The grey shaded box represents the extent of the two CNV segments. The gene content was assessed based on the University of Maryland assembly.

Durkin et al. [15] showed that colour sidedness in various cattle breeds is determined by two CNVs that duplicate a small part of the BTA6 and BTA29. Because of the strong link between white coloration and gonadal hypoplasia in Northern Finncattle and Swedish Mountain cattle, we carried out CNV analysis. A total of 2101 autosomal CNVs were identified in 94 animals. Among them a CNV segment on BTA6, extending from 71.60 Mb to 72.13 Mb and containing the *KIT* gene, was significantly associated with gonadal hypoplasia (P=5.65 x 10^-6^) (Figure 3, Figure 4A). The CNV was present in all 21 affected animals and in 52 of 73 animals of the control group. Also, a CNV segment on BTA29, extending from 19.86 Mb to 20.32 Mb, appeared to be frequent in the Northern Finncattle. The CNV segment on BTA29 was identified in 14 affected and 44 unaffected animals, but was not associated with gonadal hypoplasia (Figure S2). This segment is in the immediate vicinity of the most significantly associated gonadal hypoplasia SNPs (Figure 4B). To ensure that CNV segment on BTA6 was not associated to gonadal hypoplasia nominal p-values of the GWAS for the SNPs located vicinity of the segment were studied (Tables S4 and S5). Only with the Design D in the genotypic test a few SNPs with suggestive association was found. This design included only unaffected predominantly white and coloured animals so the found associations were related to coat colour not gonadal hypoplasia.

Durkin et al. [15] also showed that the two colour sidedness loci result from a translocation of a chromosomal segment, encompassing the *KIT* gene, from BTA6 to BTA29 and a part of this fragment back to BTA6 near the *KIT* gene. To investigate whether similar duplications are present in Northern Finncattle and the Swedish Mountain breed, we used 38 animals with copy number (CN) four on BTA6 as the case group and 21 animals with CN two as a control group to identify a potential translocation. The CN status was determined based on the array genotypes. We found a strong association on BTA29 (19,661,149 bp, P = 1.66 x 10^-28^), indicating a translocation of the BTA6 segment to BTA29 (Figure S3). We next analysed 58 and 36 animals with and without CNV on BTA29, respectively, and identified a strong association on BTA6 (72,198,048 bp, P=1.69 x 10^-14^, Figure S4), indicating a translocation from the BTA29 segment to BTA6. These positions agree with those described by Durkin et al. [15] indicating that the duplications identified are the same.

### Cytogenetic studies confirm translocations between BTA6 and BTA29

Three Northern Finncattle animals were selected for cytogenetic investigations based on the CN status of the BTA6-segment. One of these animals was affected by gonadal hypoplasia. A Western Finncattle and an Eastern Finncattle animal were also included in the comparative analysis. These breeds are not affected by the gonadal hypoplasia. Traditionally, Western Finncattle is a solid brown breed and Eastern Finncattle is a brown breed with the colour-sided phenotype. Bacterial artificial chromosome (BAC) clones specific to the CN areas of chromosomes BTA6 (red FISH signal) and BTA29 (green FISH signal) were used as probes in FISH experiments. Overlapping red and green signals appear yellow. In the Western Finncattle animal two red (BTA6 homologs) and two green (BTA29 homologs) signals were recorded. The Eastern Finncattle animal showed the following pattern: two yellow signals on BTA6 and two green signals on BTA29. All three animals from the Northern Finncattle breed showed red and yellow signals on BTA6 while the patterns on BTA29 varied. The first unaffected Northern Finncattle showed green and yellow signals on BTA29 whereas the second unaffected Northern Finncattle showed only green signals. The affected Northern Finncattle animal had yellow signals on BTA29 (Figure S5). Taken together, all animals except the solid brown Western Finncattle animal had one or several translocations associated with colour sidedness. The affected Northern Finncattle was homozygous for the Cs_29_ allele. This supports our GWAS and CNV results that showed strong association between the Cs_29_ allele and gonadal hypoplasia.

### CNV confirmation using quantitative PCR

The number of the Cs_29_ alleles was analysed by quantitative PCR in nine affected and 21 unaffected animals. The results agree with the CNV results based on the array genotypes. However, using qPCR we could not distinguish between heterozygous and homozygous animals.

### Confirmation of CNV using conventional PCR

To characterize the translocation present in Northern Finncattle and Swedish Mountain cattle we used PCR primers according to Durkin et al. [15] and one additional primer pair that flanked the insertion site of wild type BTA29. Analysed animals included also two animals that were omitted from the GWAS analyses because of the genotyping failure. Colour of these two animals was predominantly brown and white. The results showed that all affected animals were homozygous for the Cs_29_ allele and 15 animals had the Cs_6_ allele. Of the unaffected animals, 18 were homozygous and 37 were heterozygous for the Cs_29_ allele and 20 did not have the Cs_29_ allele. The Cs_6_ allele was detected from 44 control animals (Table 2). The ratio of the Cs_6_ allele is slightly higher in affected than in unaffected animals: 15/21 (71%) and 44/75 (59%) respectively. More importantly the occurrence of Cs_6_ in control animals that were homozygous for the Cs_29_ allele was 14/18 (78%) which is almost the same as in affected animals (Table 2). Like the case animals, all homozygous control animals were predominantly white. The ratio of the Cs_6_ allele in white control animals that were heterozygous for the Cs_29_ allele was 11/18 (61%) which is also quite close to the Cs_6_ ratio of the case animals (Table S6). These results suggests that the homozygous Cs_29_ allele might have pleiotropic effects on both coat colour and gonadal hypoplasia but the Cs_6_ allele affects only the coat colour. The results are in line with the CNV results based on array genotypes and qPCR. Additionally, we determined the DNA sequence of all PCR products amplified from the selected 12 animals (results not shown). The sequences were comparable with those obtained by Durkin et al. [15].

**Table 2:** The PCR analysis of CNVs.

	**Affected**			**Unaffected**		
	+/+	+/Cs29	Cs29/Cs29	+/+	+/Cs29	Cs29/Cs29
+/+	0	0	6	10	17	4
Cs6/-	0	0	15	10	20	14
total	0	0	21	20	37	18

The PCR analysis of CNVs based on primers designed by Durkin et al. [15] and us. All 96 animals were analysed including two control animals whose genotyping failed. The animals are divided according to alleles in BTA29 and BTA6. Heterozygous and homozygous carriers of the Cs_6_ allele could not be distinguished.

### A genome-wide association study comparing the case and control animals homozygous for the Cs29 allele revealed no association

Given that the Cs_29_ allele was associated with gonadal hypoplasia we performed an additional conditional GWAS where the case animals were compared with 18 control animals that were homozygous for the Cs_29_ allele. The only suggestively associated allele was the SNP on BTA23 at 52,435,290 bp which was shown to have discrepant genotypes (Figure S6).

## Discussion

The results of this study strongly suggest that the predominantly left-sided gonadal hypoplasia in Northern Finncattle and Swedish Mountain cattle is associated with a complex chromosomal rearrangement including a ~500kb duplicated segment from BTA6 which is translocated to BTA29. This duplicated segment includes the entire coding sequence of the *KIT* gene. Given that the affected animals were homozygous for the translocated duplication, our study supports the recessive disease model and highlights *KIT* gene dosage as a significant risk factor.

We showed that the translocated segment is the same as one of the two previously described translocated segments Cs_29_ and Cs_6_ that are responsible for colour sidedness in cattle [15]. The colour-sided pattern is demonstrated by variable coating from coloured animals with a white line in the back to almost white animals with coloured parts only in ears, muzzle and feet. The latter coat colour pattern is common for Northern Finncattle and Swedish Mountain cattle breeds. Settergren [6] showed that the predominantly white coat colour of Swedish Mountain cattle was strongly associated with gonadal hypoplasia. Our GWAS data support the hypothesis that the Cs_29_ allele is associated with both white coat colour and gonadal hypoplasia. We did not find any association when comparing predominantly white affected and unaffected animals. Moreover, the association signal was stronger when affected animals were compared with coloured animals than when affected animals were compared with all unaffected animals. These results are because most of the white animals carry at least one copy of the Cs_29_ allele and this implicates correlation of Cs_29_ allele with both white coat colour and gonadal hypoplasia. However, comparison of unaffected predominantly white animals and unaffected predominantly coloured animals showed no association that would have exceeded the genome-wide threshold level. This result was surprising but can be explained with the knowing that some of the unaffected predominantly coloured animals were colour sided and part of them had the Cs_29_ allele. Moreover, some of the unaffected predominantly white animals lacked the Cs_29_ allele. This indicates stronger correlation of Cs_29_ allele with gonadal hypoplasia than with predominantly white coat colour. Comparisons across the study groups strongly suggest that the Cs_29_ allele is associated with gonadal hypoplasia and not only reflects different coat colour patterns.

It is unlikely that Cs_6_ allele is related to gonadal hypoplasia given that the CNV analyses showed no association between the Cs_6_ allele and gonadal hypoplasia. Furthermore, the conventional PCR results showed that the Cs_6_ allele is only slightly enriched in affected animals when compared with all unaffected animals. More importantly, the frequency of the Cs_6_ allele was almost the same in case animals as in control animals that were homozygous for the Cs_29_ allele.

Mutations within the *KIT* gene cause white colour also in horse and pig, but there are no reports of associated gonadal hypoplasia. On the other hand, several mouse models harbouring *KIT* mutations show pleiotropic effects causing reduced fertility and white colouring. Many of these mice mutants suffer from anaemia, tumour formation and deafness (MGI:96677, http://www.informatics.jax.org/). We are not aware that the affected animals in our study had any other disease symptoms besides gonadal hypoplasia. Yet the same kind of gonadal hypoplasia, as in Northern Finncattle and Swedish Mountain cattle, has not been observed in any mouse studies. Only for the Nguni breed ( *Bos* *indicus* x *Bos taurus* cross) are there reports of a hereditary form of gonadal hypoplasia resembling that form found in Northern Finncattle and Swedish Mountain cattle [24-26]. Interestingly, the Nguni breed also presents the colour-sided phenotype.

Durkin et al. [15] implicated that loci on BTA6 and BTA29 account for most, if not all, colour sidedness in cattle. Moreover, Brenig et al. [27] showed that in White Galloway cattle and White Park cattle the predominantly white coat colour was attributed to the Cs_29_ allele. This was also shown in our study where only five animals out of 62 predominantly white animals did not have the Cs_29_ allele. Several cattle breeds have this allele in BTA29, but hereditary gonadal hypoplasia has only been reported in Northern Finncattle, Swedish Mountain cattle and Nguni cattle. This might be because the proportion of animals homozygous for the Cs_29_ allele is too small in several colour sided breeds for this type of recessive gonadal hypoplasia with incomplete penetrance to be diagnosed.

Another hypothesis for the genetic cause of the gonadal hypoplasia is that Swedish Mountain cattle and Northern Finncattle have other breed specific changes in the genome in addition to the Cs_29_ allele. This conclusion implies that Nguni might have different mutations that cause the gonadal hypoplasia. The GWAS studies showed no other reliable association between the cases and controls than the association to BTA29. However, the additional mutations might remain undetected because of the small sample size in the present study or because of the location of the mutation. If mutation is within the translocated area or in corresponding area in the original chromosome the mapping with GWAS might not work.

Our findings that all affected animals had two ectopic *KIT* genes support the postulated mode of recessive inheritance [7]. Nevertheless, 17 unaffected animals also had two ectopic *KIT* genes. This might be explained by incomplete penetrance or by the presence of an additional mutation or mutations besides the Cs_29_ allele.

In conclusion, we showed that a hereditary form of gonadal hypoplasia in Swedish Mountain cattle and Northern Finncattle is associated with homozygosity for the Cs_29_ allele. There is need for further investigations to understand fully the mechanisms causing the disorder. However, to our knowledge, this is the first report indicating a duplication of the *KIT* gene with predominantly left-sided gonadal hypoplasia in mammals.

## Materials and Methods

### Ethics statement

The blood sampling and clinical examinations were carried out following standard veterinary protocols in Finland. The Animal Ethics Committee of the State Provincial Office of Southern Finland approved all animal work (ESAVI-2010-03428/Ym-23).

### Gonad examination and sampling

Clinical examinations were done during farm visits by experienced veterinarians. Bulls and bull calves were palpated for testicle size and symmetry. Female animals older than 16 months, excluding animals more than five months pregnant, were studied by ovarian palpation per rectum. After clinical examination, 9 ml of EDTA venous blood were collected from the animals suitable for this study. Semen samples of artificial insemination bulls included in the study were also collected. From the post-mortem study animals’ gonads were examined visually, palpated and weighed. Histological and tissue samples for DNA extraction were taken from the collected gonads. Histological samples were subjected to standard Bouin’s fixation and embedded in paraffin. Sections (5 µm) were cut and stained with haematoxylin-eosin (HE).

### Animals considered affected by gonadal hypoplasia

Cows and heifers with one ovary of extremely small size were considered to be affected by gonadal hypoplasia. Usually the hypoplastic ovary was undetectable by palpation per rectum. Bulls and bull calves were considered to be affected by unilateral gonadal hypoplasia if their testicles clearly differed in size, i.e. one testicle being more than two times larger than the other testicle in young calves and for bulls’ one testicle being more than three times larger than the other testicle. Bilateral gonadal hypoplasia was diagnosed only in one bull selected for semen collection. Both testicles were very small and there were no sperm cells in the ejaculate. The testicle histology only showed Sertoli cells and no spermatogonia in seminiferous tubules in the hypoplastic testicles. When possible, the clinically diagnosed affected animals were re-examined during a second farm visit or the gonads were studied after slaughter.

### DNA extraction

Genomic DNA from blood samples was extracted by automatic isolation (Magnetic Separation Module I, Chemagen) and genomic DNA from tissue and semen samples was extracted using a commercially available Qiagen Kit (QIAamp DNA Mini Kit). Extractions of the blood and tissue samples were made according to the manufacturer’s instructions. The extraction of DNA from semen samples was made according to DNA Purification from Tissues-protocol in QIAamp DNA Mini Kit handbook with some modifications. From 200 to 500 µl of frozen semen was centrifuged for 5 min at 100 × g. The supernatant was moved and the pellet was washed with 200 µl of phosphate buffered saline (PBS). The Qiagen buffer ALT was added up to 300 µl and 20 µl of Proteinase K and dithiothreitol was also added. The mix was incubated for 1 h at 56 °C. During incubation the sample was pulse vortexed four times for 15 sec. 300 µl of Qiagen buffer AL was added and the pulse vortex repeated. The sample was incubated for 10 min at 56 °C and thereafter 150 µl of 96% alcohol was added. The sample was pulse vortexed and incubated for 3 min at room temperature. The whole mixture was applied to the QIAamp Mini spin column and centrifuged at 6,000 × g for 1 min. The filtrate was discarded and the column was washed twice with 500 µl of Qiagen buffer AW1 and once with 500 µl of Qiagen buffer AW2 (centrifugation at 6,000 × g for 1 min). The column was dried with centrifugation at 20,000 × g for 3 min. The DNA was eluted with 50 µl of distilled water, incubated for 1 min at room temperature and centrifuged at 20,000 × g for 1 min.

### Animals selected for genotyping

DNA samples from 96 animals were included in this study. Animals with total unilateral or bilateral hypoplasia were included in the case group that comprised 21 animals (10 males and 11 females). Of these animals, one was affected with bilateral, 18 with left-sided and two with right-sided gonadal hypoplasia. The control group included 75 unaffected animals. Among the studied animals 91 were Northern Finncattle and five were Swedish Mountain cattle (two cases and three controls).

### High-density genotyping and quality control

Ninety-six animals (21 affected / 75 unaffected) were genotyped with the Illumina BovineHD Bead chip interrogating genotypes of 777,962 SNPs. Genotype calling was performed using default parameters of Illumina’s *BeadStudio*. Quality control was carried out with *PLINK* v1.07 [28]. We excluded 1224, 343 and 1735 SNPs with Y-chromosomal, mitochondrial and unknown chromosomal position, respectively, for further analysis. The genotypes of two unaffected animals were omitted because genotyping failed in more than 10% of the SNPs. We further excluded 6229 SNPs because genotyping failed in more 10% of the individuals and 121,657 monomorphic SNPs. The final dataset comprised 94 animals and 647,971 SNPs with an average call-rate of 99.67%. The chromosomal position of the SNPs was determined based on the UMD3.1 assembly of the bovine genome [29].

### Genome-wide association study

Fisher exact tests of allelic and genotypic associations were performed to compare genotypes in cases vs. controls at each SNP in turn using *PLINK* [28]. We considered SNPs with P < 7.71 x 10^-8^ as significantly associated (Bonferroni-corrected threshold for multiple testing). Quantile-quantile plots were inspected and genomic inflation factors were calculated according to Devlin and Roeder [30] to assess the extent of false positive association signals.

### Detection of copy number variants

Genotype signal intensities obtained from genotyping with the Illumina BovineHD Bead chip (see above) were analysed with *PennCNV* [31] to identify copy number variations (CNV). Briefly, the implemented CNV-detection algorithm considers both the log R ratio (LRR) and the B allele frequency (BAF), as well as the allele frequency and the distance of adjacent SNPs. Two individuals and 6229 SNPs with poor genotyping quality (see above) were not considered for the identification of CNVs. The presence of CNVs containing a minimum number of 10 SNPs corresponding to a minimum length of approximately 35 Kb was compared in cases vs. controls using Fisher exact tests.

### Cytogenetic analysis

Metaphase spreads were obtained from short-term lymphocyte cultures, established for five animals. The FISH study was carried out according to a protocol described by Durkin et al. [15]. Two BAC clones (RP42-160M9, RP42-156I13) covering the region of interest on chromosome BTA6 (region 72,566,605–72,817,995bp, UMD 3.0) and two BAC clones (RP42-37P11, RP42-116G8) covering the region of interest on BTA29 (region 20,772,406–21,035,251bp, UMD 3.0) were derived from the RPCI-42 Bovine BAC Library (http://bacpac.chori.org/home.htm). BAC DNA was isolated using an alkaline lysis method and labelled by random priming. The isolated DNAs from two BAC clones specific to BTA6 were mixed equally and labelled using biotin-11-dUTP, while DNA from two BAC clones specific for BTA29 was also mixed and labelled with digoxigenin-11-dUTP. The labelled probes with an excess of bovine Cot-1 DNA were separately denatured for 10 min at 70°C and applied on denatured chromosome slides. Hybridization was carried out overnight at 37°C. After slide washing, biotin-labelled probes were detected using streptavidin-Cy3 (Amersham, 1:200, red colour) and digoxigenin-labelled probes were detected with antidigoxigenin-fluorescein Fab fragments (Roche, 1:200, green colour). The slides were counterstained with Vectashield containing DAPI (Vector Laboratories) and examined with an epifluorescence Nikon E600 Eclipse microscope equipped with a cooled digital CCD camera and Lucia software.

### QPCR

QPCR was used to validate CNV identified after bioinformatics analysis. Altogether 30 samples were analysed: nine cases and 21 controls. A simple method based on Weksber et al. [32] and Lachman et al. [33] was applied for analysis. Two primer pairs designed using Primer 3 [34] were located in the *KIT* region: precisely in exon 8 (GGGCCAGTGGATGTACAGAT) and 9 (TGCAAAGTTAAAAGAGGCAGA); the second pair in exon 18 (CACATTTGAAAGTGATGTCTGG) and 19 (AGAACTTAGAATCGACTGGCATT). 10 µl of QPCR reaction was composed from the Fast SYBR® Green Master Mix (Life Technologies) and 2.5 pmol of each primer. All amplifications were carried out in Applied Biosystems 7500 Fast Real-Time PCR System (Life Technologies) according to manufacturer’s recommendations. *H6PD* was used as a reference gene; both primers were located in the first exon (AAGGTCCTGGAGTCCCTGTC, GTAGAAAATTCGGCCGGTCT).

### PCR and sequencing

All animals were analysed with standard PCR carried out using breakpoint primers designed by Durkin et al. (Table 2) [15] and one additional primer pair PSK_α-β2_R (TGGGTAGACAGGTTTGTTTCC and TCTTGACCACTTGCATTGGA) that flanked the insertion site of the wild type BTA29. A PCR reaction of 20 µl volume containing 20 ng genomic DNA, 1x Qiagen PCR buffer, 1.5 mM MgCl2, 200 µM of each nucleotide, 5 pmol of each forward and reverse primer (Sigma) and 0.5 units of Taq Polymerase (Qiagen) was performed under the following conditions: initial denaturing at 95 °C for 3 min, followed by 35 cycles at 94 °C for 30 sec, 60 °C for 1 min, 72 °C for one minute and the final extension at 72 °C for 3 min. PCR products were visualized together with GeneRulerTM100bp DNA Ladder (Fermentas, Thermo Scientific) on 1.5% agarose gel.

Five case samples, two control samples with a duplication of the BTA6 segment, two control samples with both studied duplications and three control samples without the duplications were subjected to sequencing. A 10-20 ng of purified PCR product was mixed with 0.5 µl BigDye® Terminator v3.1 (Life Technologies) and 0.5 µl of the forward or reverse PCR primer (2.5 pmol). The sequencing reaction was performed under the following conditions: initial denaturing at 96 °C for 10 sec, followed by 35 cycles, including 10 sec in 96 °C, 5 sec in 50 °C, and 4 min in 60 °C. The gel filtration of the sequencing reaction was applied using the MultiScreen filtration plate (MAHVN4510; Millipore) and Sephadex G-50 Fine (Sigma) followed by capillary electrophoresis carried out on 3130xl Genetic Analyzer (Life Technologies). Base calling, sequence alignment and polymorphism detection were made using the Phred/Phrap/Polyphred software [35-37]. Sequences were inspected using Consed [38].

Seven cases and ten controls were sequenced (see above) with primers CAATTTTAAGCATGTGCTGAGG and ACAGCCTCTGGTCTGTCTGG to validate the genotype calls for the SNP on BTA23 at 52,435,290 bp.

## Supporting Information

Figure S1 **Examples of the common colour pattern in Northern Finncattle.** Most commonly, Northern Finncattle is almost white with black or brown in ears and muzzle. The flanks and legs can also be partly coloured or spotted.
(TIF)

Figure S2 **Average log R ratio of animals carrying the ectopic BTA29 segment.** The average log R ratio was calculated from 15 affected and 44 unaffected animals that carry the duplicated segment of BTA29. The 5-SNP-sliding window log R ratio is presented for 563 SNPs.
(PNG)

Figure S3 **Localisation of a translocation of a *KIT* containing segment to chromosome 29.** Animals carrying two and four copies of the BTA6 segment were compared using Fisher exact tests. The red dots represent significantly associated SNPs (P < 7.71 x 10^-8^).
(EPS)

Figure S4 **Localisation of a translocation of a BTA29 segment to chromosome 6.** Animals with and without the presence of a BTA29 CNV were compared using Fisher exact tests. The red dots represent significantly associated SNPs (P < 7.71 x 10^-8^).
(EPS)

Figure S5 **FISH studies.** Three Northern Finncattle (NFC) animals with different combinations of the Cs_29_ allele and one animal of the Western Finncattle (WFC) and Eastern Finncattle (EFC) were analysed by FISH with two BAC probes. The Cs_29_ allele is associated with both colour sidedness and gonadal hypoplasia and it corresponds to the red FISH signal or the red bar. The Cs_6_ allele is associated with colour sidedness and corresponds to the green FISH signal or the green bar. Overlapping red and green signals appear yellow. All animals except the solid brown Western Finncattle had one or several Cs alleles. The animal NFC 164 is affected with gonadal hypoplasia and it is homozygous for the Cs_29_ allele.
(TIF)

Figure S6 **Association of 647,971 SNPs with the affection status of 39 animals homozygous for the Cs_29_ allele.** Association analysis was performed using Fisher exact tests of allelic association for 21 affected and 18 unaffected animals homozygous for the Cs_29_ allele.
(JPG)

Table S1 **The association between the proportion of coat pigmentation and total (unilateral or bilateral) gonadal hypoplasia in the Swedish Mountain breed females (modified from Settergren [6]).**
(DOCX)

Table S2 **Nominal p-values for the SNPs significantly associated to gonadal hypoplasia in BTA29 (allelic test).** P-values were calculated with Fisher exact tests in PLINK to determine allelic association in four different case-control cohorts (Table 1).
(XLSX)

Table S3 **Nominal p-values for the SNPs significantly associated to gonadal hypoplasia in BTA29 (genotypic test).** P-values were obtained using a 2df genotypic test implemented in PLINK to determine association in four different case-control cohorts (Table 1).
(XLSX)

Table S4 **Nominal p-values for the SNPs located between 71502659 bp and 71990541 bp in BTA6 (allelic test).** P-values were calculated with Fisher exact tests in PLINK to determine allelic association in four different case-control cohorts (Table 1).
(XLS)

Table S5 **Nominal p-values for the SNPs located between 71502659 bp and 71990541 bp in BTA6 (genotypic test).** P-values were obtained using a 2df genotypic test implemented in PLINK to determine association in four different case-control cohorts (Table 1).
(XLS)

Table S6 **Predominantly white coloured animals divided according to affection status and alleles in BTA6 and BTA29.** The CNVs were studied with PCR and primers designed by Durkin et al. [15] and us. Heterozygous and homozygous carriers of the Cs_6_ allele could not be distinguished.
(DOCX)

## References

[1] Romagnoli S, Schlafer DH (2006) Disorders of sexual differentiation in puppies and kittens: A diagnostic and clinical approach. Vet Clin North Am Small Anim Pract 36: 573-606. doi:10.1016/j.cvsm.2005.12.007. PubMed: 16564415. 16564415 10.1016/j.cvsm.2005.12.007

[2] Palmieri C, Schiavi E, Della Salda L (2011) Congenital and acquired pathology of ovary and tubular genital organs in ewes: A review. Theriogenology 75: 393-410. doi:10.1016/j.theriogenology.2010.09.020. PubMed: 21111461. 21111461 10.1016/j.theriogenology.2010.09.020

[3] Mäkinen A, Hasegawa T, Mäkilä M, Katila T (1999) Infertility in two mares with XY and XXX sex chromosomes. Equine Vet J 31: 346-349. doi:10.1111/j.2042-3306.1999.tb03829.x. PubMed: 10454097. 10454097 10.1111/j.2042-3306.1999.tb03829.x

[4] Abe S, Miyake YI, Kageyama SI, Watanabe G, Taya K et al. (1999) Deletion of the sry region on the Y chromosome detected in a case of equine gonadal hypoplasia (XY female) with abnormal hormonal profiles. Equine Vet J 31: 336-338. PubMed: 10454094. 10454094

[5] Bidarkar SS, Hutson JM (2005) Evaluation and management of the abnormal gonad. Semin Pediatr Surg 14: 118-123. doi:10.1053/j.sempedsurg.2005.01.008. PubMed: 15846569. 15846569 10.1053/j.sempedsurg.2005.01.008

[6] Settergren I (1964) The ovarian morphology in clinical bovine gonadal hypoplasia with some aspects of its endogrine relations. Stockh Almqvist Wiksells: 108.

[7] Eriksson K (1943) Hereditary forms of sterility in cattle. biological and genetical investigations. Lund Håkan Ohlssons Boktryckeri: 155.

[8] Lagerlöf N, Settergren I (1961) Gonadenhypoplasie beim rind der schwedischen gebirgsrasse. Zuchthygiene 5: 141-158.

[9] Lagerlof N, Settergren I (1953) Results of 17 years control of hereditary ovarian hypoplasia in cattle of the swedish highland breed. Cornell Vet 43: 52-64. PubMed: 13010079. 13010079

[10] Lagerlof N, Boyd H (1953) Ovarian hypoplasia and other abnormal conditions in the sexual organs of cattle of the swedish highland breed - results of post-mortem examination of over 6,000 cows. Cornell Vet 43: 64-79. PubMed: 13010080. 13010080

[11] Tarbashevich K, Raz E (2010) The nuts and bolts of germ-cell migration. Curr Opin Cell Biol 22: 715-721. doi:10.1016/j.ceb.2010.09.005. PubMed: 20947321. 20947321 10.1016/j.ceb.2010.09.005

[12] Richardson BE, Lehmann R (2010) Mechanisms guiding primordial germ cell migration: Strategies from different organisms. Nat Rev Mol Cell Biol 11: 37-49. doi:10.1038/nrm2815. PubMed: 20027186. 20027186 10.1038/nrm2815 PMC4521894

[13] Olson T (1999) Genetics of colour variation. In: Fries R Ruvinsky A The Genetics of Cattle. Wallingford: CABI Publishing pp. 33-51.

[14] Wriedt C (1925) Colorsided cattle: Some remarks concerning their occurrence and heredity. J Hered 16: 51-56.

[15] Durkin K, Coppieters W, Drögemüller C, Ahariz N, Cambisano N et al. (2012) Serial translocation by means of circular intermediates underlies colour sidedness in cattle. Nature 482: 81-84: 81–4. doi:10.1038/nature10757. PubMed: 22297974 Retrieved onpublished at whilst December year 1111 from . doi:10.1038/nature10757 22297974 10.1038/nature10757

[16] Bernex F, De Sepulveda P, Kress C, Elbaz C, Delouis C et al. (1996) Spatial and temporal patterns of c-kit-expressing cells in WlacZ/+ and WlacZ/WlacZ mouse embryos. Development 122: 3023-3033. PubMed: 8898216. 8898216 10.1242/dev.122.10.3023

[17] Buehr M, McLaren A, Bartley A, Darling S (1993) Proliferation and migration of primordial germ cells in we/we mouse embryos. Dev Dyn 198: 182-189. doi:10.1002/aja.1001980304. PubMed: 8136523. 8136523 10.1002/aja.1001980304

[18] Nishikawa S, Kusakabe M, Yoshinaga K, Ogawa M, Hayashi S et al. (1991) In utero manipulation of coat color formation by a monoclonal anti-c-kit antibody: Two distinct waves of c-kit-dependency during melanocyte development. EMBO J 10: 2111-2118. PubMed: 1712289. 1712289 10.1002/j.1460-2075.1991.tb07744.x PMC452897

[19] Ogawa M, Nishikawa S, Yoshinaga K, Hayashi S, Kunisada T et al. (1993) Expression and function of c-kit in fetal hemopoietic progenitor cells: Transition from the early c-kit-independent to the late c-kit-dependent wave of hemopoiesis in the murine embryo. Development 117: 1089-1098. PubMed: 7686845. 7686845 10.1242/dev.117.3.1089

[20] Marklund S, Kijas J, Rodriguez-Martinez H, Rönnstrand L, Funa K et al. (1998) Molecular basis for the dominant white phenotype in the domestic pig. Genome Res 8: 826-833. PubMed: 9724328. 9724328 10.1101/gr.8.8.826 PMC310759

[21] Johansson Moller M, Chaudhary R, Hellmén E, Höyheim B, Chowdhary B et al. (1996) Pigs with the dominant white coat color phenotype carry a duplication of the KIT gene encoding the mast/stem cell growth factor receptor. Mamm Genome 7: 822-830. doi:10.1007/s003359900244. PubMed: 8875890. 8875890 10.1007/s003359900244

[22] Haase B, Brooks SA, Tozaki T, Burger D, Poncet PA et al. (2009) Seven novel KIT mutations in horses with white coat colour phenotypes. Anim Genet 40: 623-629. doi:10.1111/j.1365-2052.2009.01893.x. PubMed: 19456317. 19456317 10.1111/j.1365-2052.2009.01893.x

[23] Lauvergne JJ (1970) Gonadal hypoplasia and white coat color in swedish highland cattle. J Hered 61: 43-44. PubMed: 5480937. 5480937 10.1093/oxfordjournals.jhered.a108031

[24] Kay GW, Grobbelaar JA, Hattingh J (1992) Heritable testicular hypoplasia in nguni (bos indicus) bulls: Vascular characteristics and testosterone production. J Reprod Fertil 96: 537-547. doi:10.1530/jrf.0.0960537. PubMed: 1339834. 1339834 10.1530/jrf.0.0960537

[25] Kay GW, Meyer EHH (1985) Testicular compensation in nguni (bos indicus, sanga) bulls with unilateral gonadal hypoplasia and aplasia. S Afr J Anim Sci 15: 58-60.

[26] Pretorius A, Osbourn DE (1979) Observations on hypoplasia in nguni cattle. Die Afrikanerbees joernaal = The Afrikaner cattle journal 25: 40-47.

[27] Brenig B, Beck J, Floren C, Bornemann-Kolatzki K, Wiedemann I et al. (2013) Molecular genetics of coat colour variations in white galloway and white park cattle. Anim Genet, 44: 450–3. doi:10.1111/age.12029. 2/19/2013 PubMed : 23418861 23418861 10.1111/age.12029

[28] Purcell S, Neale B, Todd-Brown K, Thomas L, Ferreira MA et al. (2007) PLINK: A tool set for whole-genome association and population-based linkage analyses. Am J Hum Genet 81: 559-575. doi:10.1086/519795. PubMed: 17701901. 17701901 10.1086/519795 PMC1950838

[29] Zimin AV, Delcher AL, Florea L, Kelley DR, Schatz MC et al. (2009) A whole-genome assembly of the domestic cow, bos taurus. Genome Biol 10: R42. doi:10.1186/gb-2009-10-4-r42. PubMed: 19393038. 19393038 10.1186/gb-2009-10-4-r42 PMC2688933

[30] Devlin B, Roeder K (1999) Genomic control for association studies. Biometrics 55: 997-1004. doi:10.1111/j.0006-341X.1999.00997.x. PubMed: 11315092. 11315092 10.1111/j.0006-341x.1999.00997.x

[31] Wang K, Li M, Hadley D, Liu R, Glessner J et al. (2007) PennCNV: An integrated hidden markov model designed for high-resolution copy number variation detection in whole-genome SNP genotyping data. Genome Res 17: 1665-1674. doi:10.1101/gr.6861907. PubMed: 17921354. 17921354 10.1101/gr.6861907 PMC2045149

[32] Weksberg R, Hughes S, Moldovan L, Bassett AS, Chow EW et al. (2005) A method for accurate detection of genomic microdeletions using real-time quantitative PCR. BMC Genomics 6: 180. doi:10.1186/1471-2164-6-180. 10.1186/1471-2164-6-180 PMC1327677 16351727

[33] Lachman HM, Pedrosa E, Petruolo OA, Cockerham M, Papolos A et al. (2007) Increase in GSK3beta gene copy number variation in bipolar disorder. Am J Med Genet B Neuropsychiatr Genet 144B: 259-265. doi:10.1002/ajmg.b.30498. PubMed: 17357145. 17357145 10.1002/ajmg.b.30498

[34] Rozen S, Skaletsky H (2000) Primer3 on the WWW for general users and for biologist programmers. Methods Mol Biol 132: 365-386. PubMed: 10547847. 10547847 10.1385/1-59259-192-2:365

[35] Ewing B, Green P (1998) Base-calling of automated sequencer traces using phred. II. error probabilities. Genome Res 8: 186-194. PubMed: 9521922. 9521922

[36] Ewing B, Hillier L, Wendl MC, Green P (1998) Base-calling of automated sequencer traces using phred. I. accuracy assessment. Genome Res 8: 175-185. doi:10.1101/gr.8.3.175. PubMed: 9521921. 9521921 10.1101/gr.8.3.175

[37] Nickerson DA, Tobe VO, Taylor SL (1997) PolyPhred: Automating the detection and genotyping of single nucleotide substitutions using fluorescence-based resequencing. Nucleic Acids Res 25: 2745-2751. doi:10.1093/nar/25.14.2745. PubMed: 9207020. 9207020 10.1093/nar/25.14.2745 PMC146817

[38] Gordon D, Abajian C, Green P (1998) Consed: A graphical tool for sequence finishing. Genome Res 8: 195-202. doi:10.1101/gr.8.3.195. PubMed: 9521923. 9521923 10.1101/gr.8.3.195

