# Supplementary figures and images for: Ectopic KIT Copy Number Variation Underlies Impaired Migration of Primordial Germ Cells Associated with Gonadal Hypoplasia in Cattle (Bos taurus)
Source: PLoS One. 2013 Sep 26;8(9):e75659. doi: 10.1371/journal.pone.0075659 (PMC3784456; doi:10.1371/journal.pone.0075659)

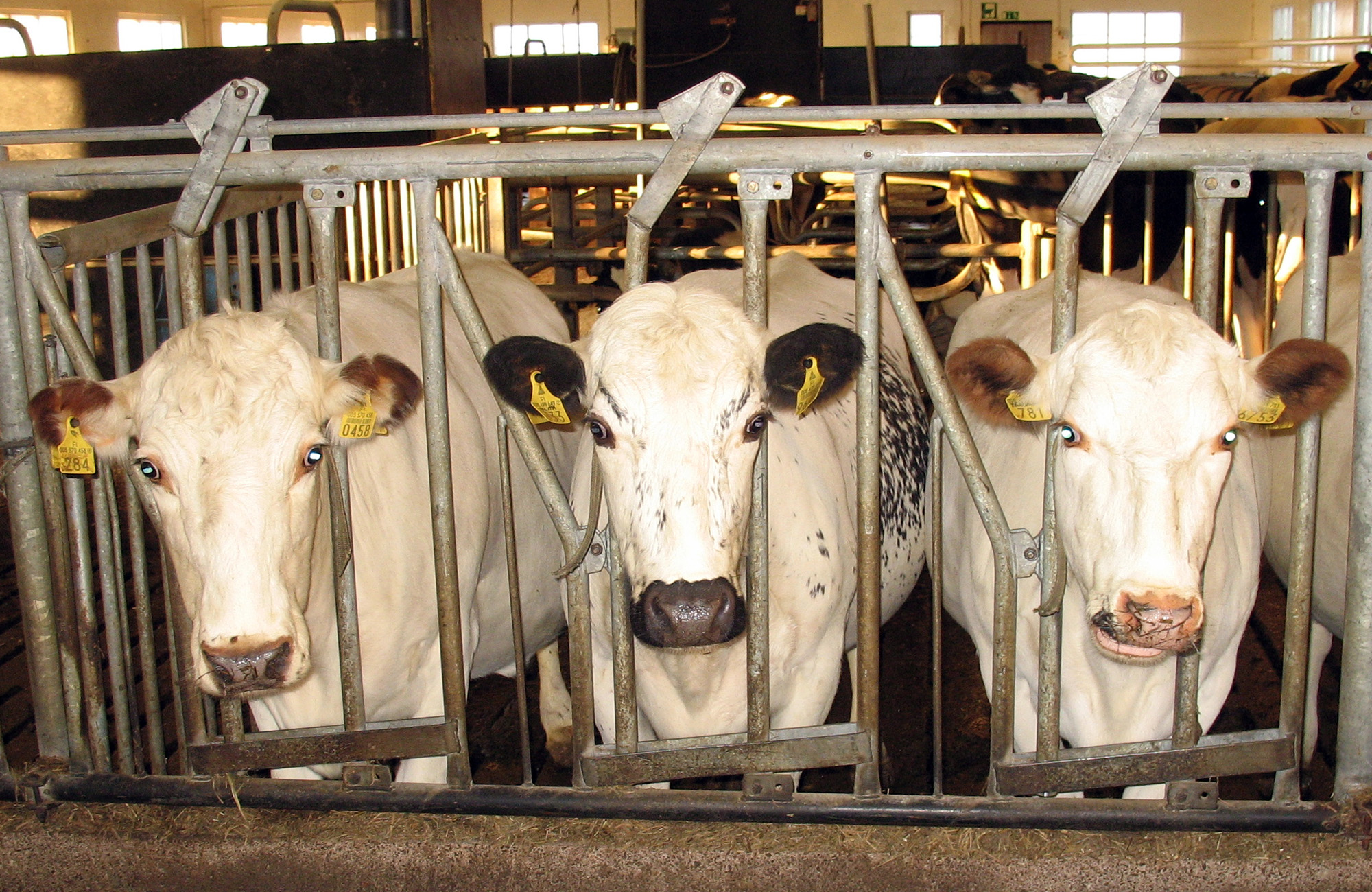

Supplement: Figure S1 — Examples of the common colour pattern in Northern Finncattle. Most commonly, Northern Finncattle is almost white with black or brown in ears and muzzle. The flanks and legs can also be partly coloured or spotted. (TIF) [file pone.0075659.s001.tif]

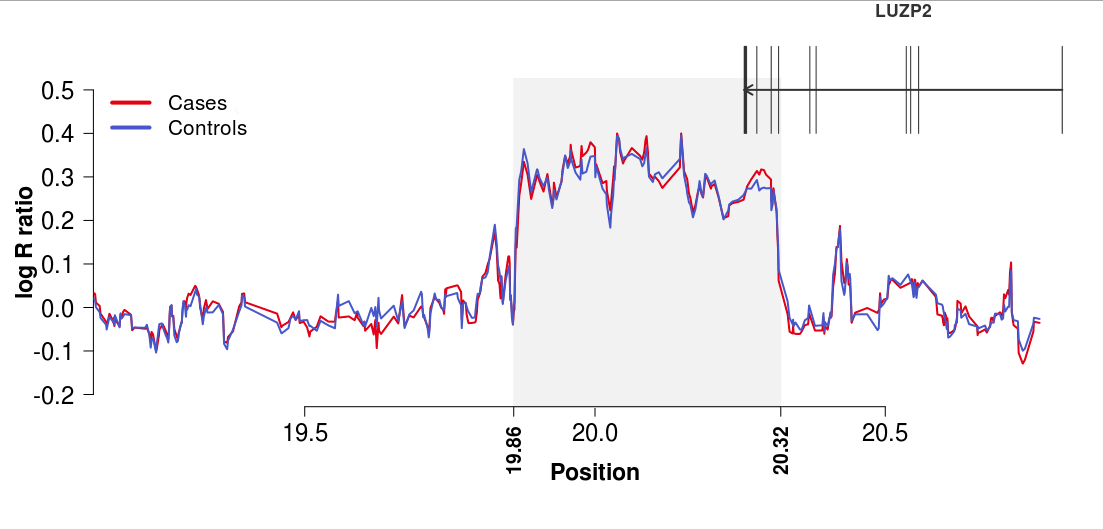

Supplement: Figure S2 — Average log R ratio of animals carrying the ectopic BTA29 segment. The average log R ratio was calculated from 15 affected and 44 unaffected animals that carry the duplicated segment of BTA29. The 5-SNP-sliding window log R ratio is presented for 563 SNPs. (PNG) [file pone.0075659.s002.png]

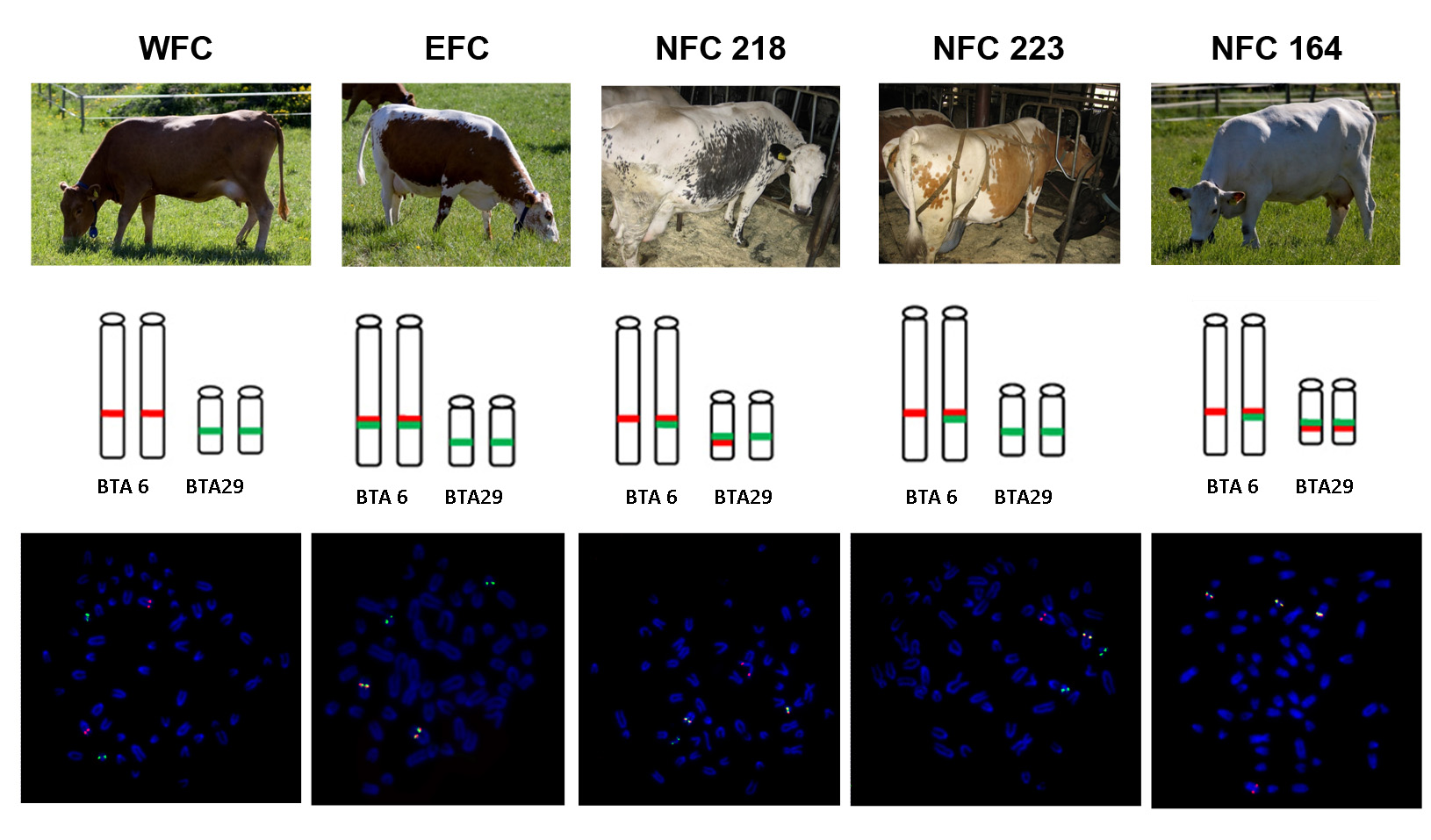

Supplement: Figure S5 — FISH studies. Three Northern Finncattle (NFC) animals with different combinations of the Cs29 allele and one animal of the Western Finncattle (WFC) and Eastern Finncattle (EFC) were analysed by FISH with two BAC probes. The Cs29 allele is associated with both colour sidedness and gonadal hypoplasia and it corresponds to the red FISH signal or the red bar. The Cs6 allele is associated with colour sidedness and corresponds to the green FISH signal or the green bar. Overlapping red and green signals appear yellow. All animals except the solid brown Western Finncattle had one or several Cs alleles. The animal NFC 164 is affected with gonadal hypoplasia and it is homozygous for the Cs29 allele. (TIF) [file pone.0075659.s005.tif]

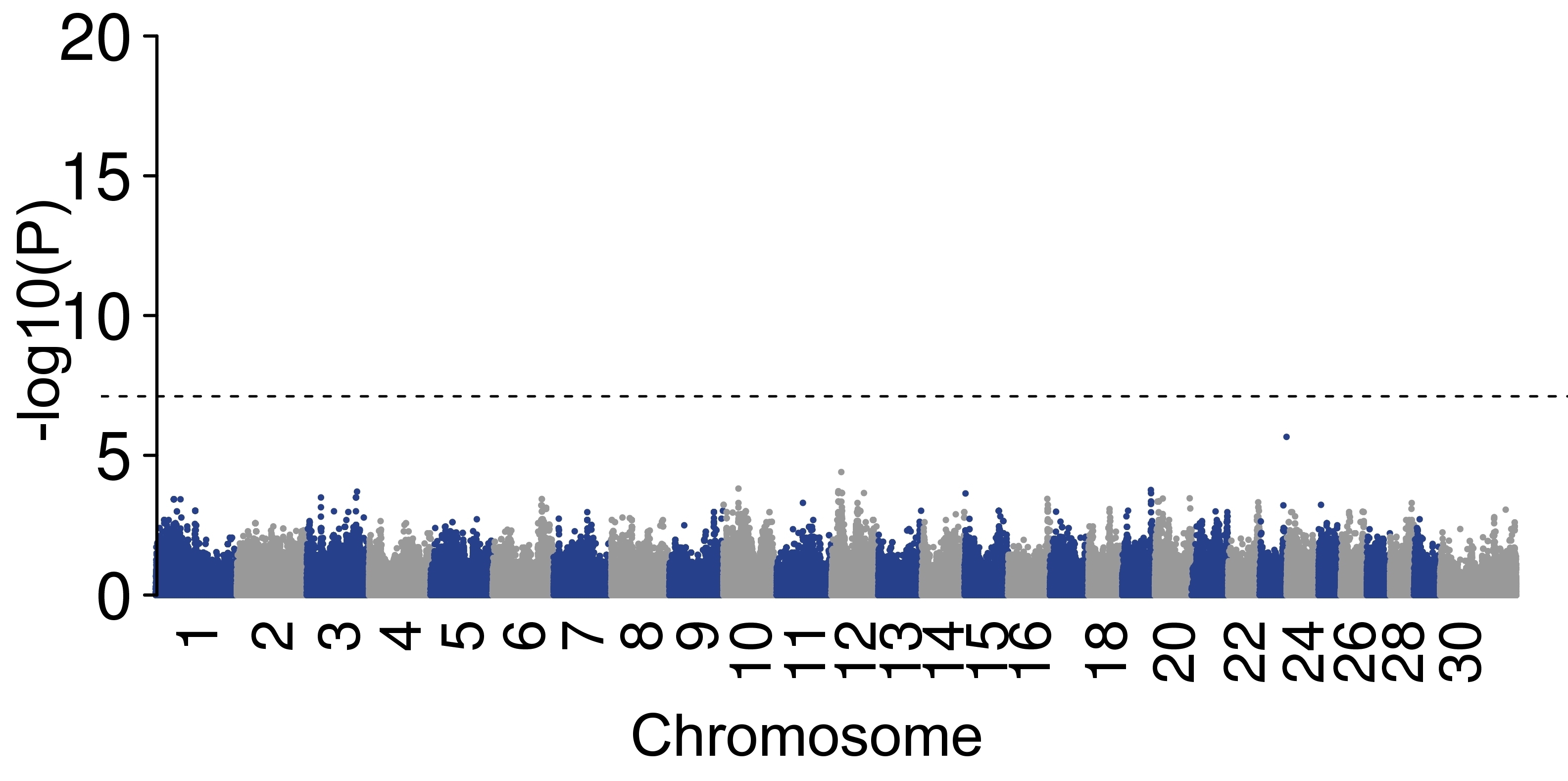

Supplement: Figure S6 — Association of 647,971 SNPs with the affection status of 39 animals homozygous for the Cs29 allele. Association analysis was performed using Fisher exact tests of allelic association for 21 affected and 18 unaffected animals homozygous for the Cs29 allele. (JPG) [file pone.0075659.s006.jpg]
