# Supplementary material for: Ectopic KIT Copy Number Variation Underlies Impaired Migration of Primordial Germ Cells Associated with Gonadal Hypoplasia in Cattle (Bos taurus)
Source: PLoS One. 2013 Sep 26;8(9):e75659. doi: 10.1371/journal.pone.0075659 (PMC3784456; doi:10.1371/journal.pone.0075659)
Supplement: Table S1 — The association between the proportion of coat pigmentation and total (unilateral or bilateral) gonadal hypoplasia in the Swedish Mountain breed females (modified from Settergren [6]). (DOCX) [file pone.0075659.s007.docx]

**Table S1.** The association between the proportion of coat pigmentation and total (unilateral or bilateral) gonadal hypoplasia in the Swedish Mountain breed females (modified from Settergren [6])

| Ovarian development | 80 – 100% white colour | 60 – 80% white colour | 30 – 60% white colour | 0 – 30% white colour | Number of animals |
| --- | --- | --- | --- | --- | --- |
| Normal | 4366 animals | 1686 animals | 573 animals | 459 animals | 7084 |
| Total hypoplasia | 618 animals | 2 animals | 0 animals | 0 animals | 620 |
| Incidence | 12.4% | 0.12% | 0% | 0% |  |
