# Supplementary material for: Ectopic KIT Copy Number Variation Underlies Impaired Migration of Primordial Germ Cells Associated with Gonadal Hypoplasia in Cattle (Bos taurus)
Source: PLoS One. 2013 Sep 26;8(9):e75659. doi: 10.1371/journal.pone.0075659 (PMC3784456; doi:10.1371/journal.pone.0075659)
Supplement: Table S6 — Predominantly white coloured animals divided according to affection status and alleles in BTA6 and BTA29. The CNVs were studied with PCR and primers designed by Durkin et al. [15] and us. Heterozygous and homozygous carriers of the Cs6 allele could not be distinguished. (DOCX) [file pone.0075659.s012.docx]

**Table S6.** Predominantly white coloured animals divided according to affection status and alleles in BTA6 and BTA29.

|  | Affected | | | Unaffected white | | |
| --- | --- | --- | --- | --- | --- | --- |
|  | +/+ | +/Cs29 | Cs29/Cs29 | +/+ | +/Cs29 | Cs29/Cs29 |
| +/+ | 0 | 0 | 6 | 4 | 7 | 4 |
| Cs6/- | 0 | 0 | 15 | 1 | 11 | 14 |
| total | 0 | 0 | 21 | 5 | 18 | 18 |
